# [2-(1-{2-[Aza­nid­yl(ethyl­sulfan­yl)methyl­idene-κ*N*]hydrazin-1-yl­idene-κ*N* ^1^}eth­yl)phenolato-κ*O*](pyridine-κ*N*)nickel(II)

**DOI:** 10.1107/S1600536812026177

**Published:** 2012-06-16

**Authors:** Reza Takjoo, Seik Weng Ng, Edward R. T. Tiekink

**Affiliations:** a Department of Chemistry, School of Sciences, Ferdowsi University of Mashhad, 91775-1436 Mashhad, Iran; b Department of Chemistry, University of Malaya, 50603 Kuala Lumpur, Malaysia; c Chemistry Department and Faculty of Science, King Abdulaziz University, PO Box 80203 Jeddah, Saudi Arabia

## Abstract

The Ni^II^ atom in the title complex, [Ni(C_11_H_13_N_3_OS)(C_5_H_5_N)], exists within a square-planar N_3_O donor set provided by *N*,*N*′,*O* atoms of the dianionic tridentate ligand and a pyridine N atom. The maximum deviation from the ideal geometry is seen in the N—Ni—N five-membered chelate bite angle of 83.28 (12)°. The pyridine mol­ecule forms a dihedral angle of 44.43 (6)° with the N_3_O donor set. Supra­molecular stacks along the *a* axis mediated by alternating π–π inter­actions between the pyridine and five- [centroid–centroid distance = 3.4784 (16) Å] and six-membered [3.4633 (17) Å] chelate rings, feature in the crystal packing.

## Related literature

For the complexing ability of *S*-alkyl esters of thio­semicarbazone derivatives, see: Ahmadi *et al.* (2012 ▶). For medicinal applications of thio­semicarbazone, see: Dilworth & Hueting (2012 ▶). For a related structure, see: Guveli & Ulkuseven (2011 ▶).
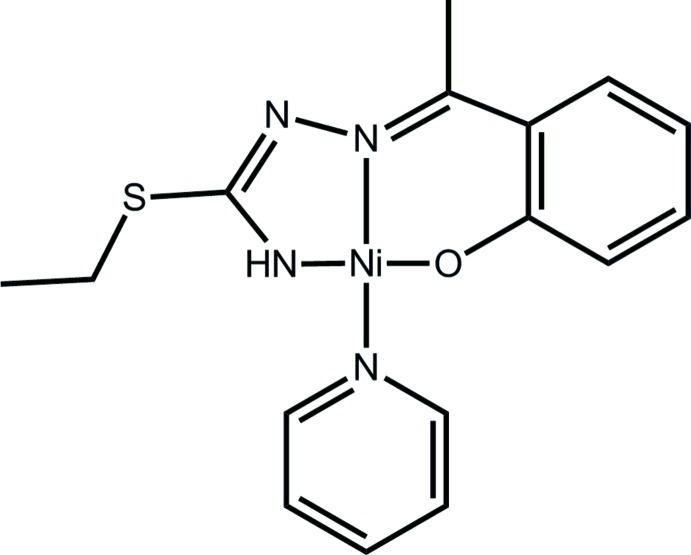


## Experimental

### 

#### Crystal data

[Ni(C_11_H_13_N_3_OS)(C_5_H_5_N)]
*M* *_r_* = 373.11
Orthorhombic, 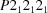

*a* = 7.2956 (4) Å
*b* = 9.8463 (5) Å
*c* = 21.7489 (11) Å
*V* = 1562.33 (14) Å^3^
*Z* = 4
Mo *K*α radiation
μ = 1.39 mm^−1^
*T* = 100 K
0.35 × 0.10 × 0.05 mm

#### Data collection

Agilent SuperNova Dual diffractometer with an Atlas detector
Absorption correction: multi-scan (*CrysAlis PRO*; Agilent, 2012 ▶) *T*_min_ = 0.790, *T*_max_ = 1.000
6002 measured reflections
3584 independent reflections
3130 reflections with *I* > 2σ(*I*)
*R*_int_ = 0.037

#### Refinement

*R*[*F* ^2^ > 2σ(*F* ^2^)] = 0.040
*wR*(*F* ^2^) = 0.075
*S* = 1.00
3584 reflections
213 parameters
1 restraint
H atoms treated by a mixture of independent and constrained refinement
Δρ_max_ = 0.45 e Å^−3^
Δρ_min_ = −0.39 e Å^−3^
Absolute structure: Flack (1983 ▶), 1501 Friedel pairs
Flack parameter: −0.028 (16)

### 

Data collection: *CrysAlis PRO* (Agilent, 2012 ▶); cell refinement: *CrysAlis PRO*; data reduction: *CrysAlis PRO*; program(s) used to solve structure: *SHELXS97* (Sheldrick, 2008 ▶); program(s) used to refine structure: *SHELXL97* (Sheldrick, 2008 ▶); molecular graphics: *ORTEP-3* (Farrugia, 1997 ▶) and *DIAMOND* (Brandenburg, 2006 ▶); software used to prepare material for publication: *publCIF* (Westrip, 2010 ▶).

## Supplementary Material

Crystal structure: contains datablock(s) global, I. DOI: 10.1107/S1600536812026177/hb6842sup1.cif

Structure factors: contains datablock(s) I. DOI: 10.1107/S1600536812026177/hb6842Isup2.hkl

Additional supplementary materials:  crystallographic information; 3D view; checkCIF report

## Figures and Tables

**Table 1:** Selected bond lengths (Å)

Ni—O1	1.828 (2)
Ni—N1	1.861 (2)
Ni—N3	1.845 (3)
Ni—N4	1.918 (2)

**Fig. 1.**
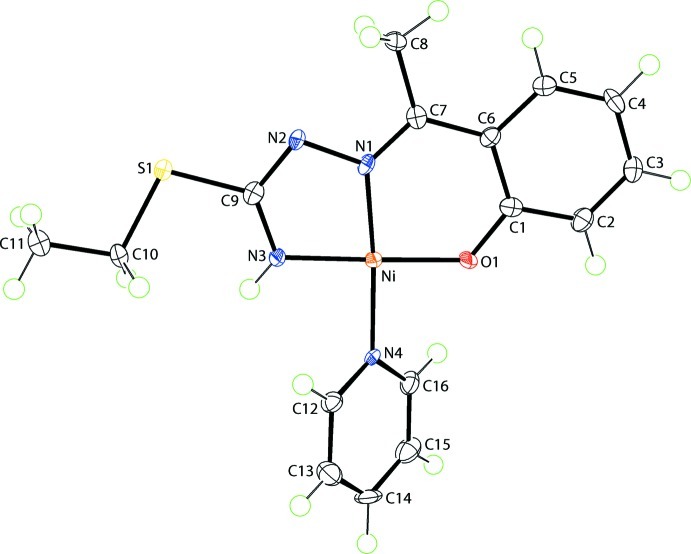
The molecular structure of (I) showing displacement ellipsoids at the 70% probability level.

**Fig. 2.**
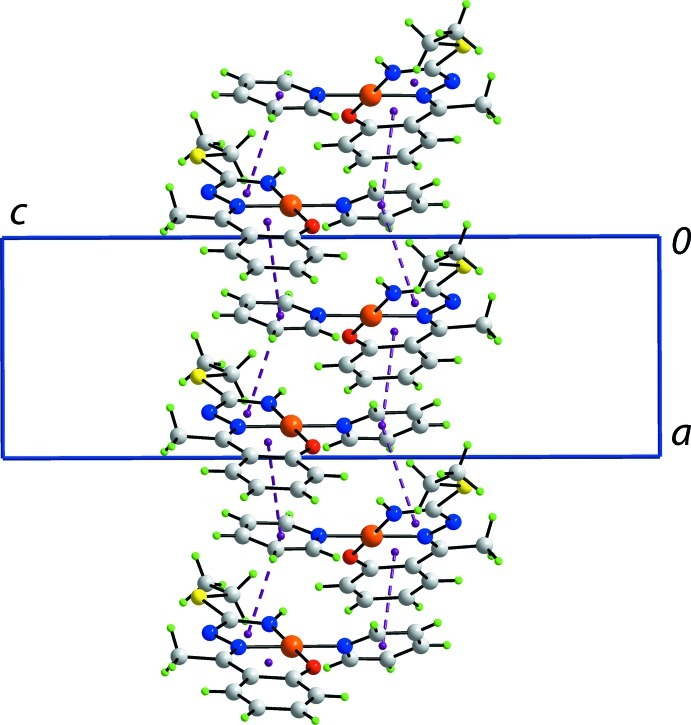
Supramolecular stack along the a axis in (I) mediated by π—π interactions, shown as purple dashed lines.

**Fig. 3.**
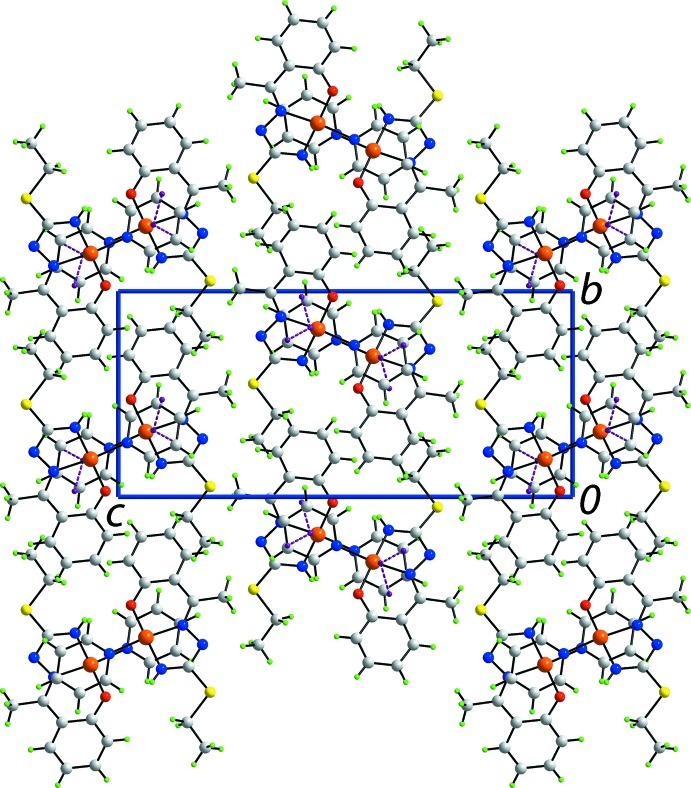
A view of the unit-cell contents of (I) in projection down the a axis. The π—π interactions are shown as purple dashed lines.

## supplementary crystallographic information

### Comment

Schiff bases derived from *S*-alkyl esters of thiosemicarbazone comprise an important class of ligands containing sulfur-nitrogen donor atoms for metals. Thus, they are capable of reacting with both transition and some main group metals (Ahmadi *et al.*, 2012) and may be used as therapeutic and imaging agents (Dilworth & Hueting, 2012). Herein, the crystal and molecular structure of the title complex, (I), is described.

The Ni^II^ atom in (I), Fig. 1, exists within a square planar N_3_O donor set defined by the *N*,*N*,*O* atoms of the dinegative tridentate ligand and a pyridine-*N* atom, Table 1. The donor set is planar with a r.m.s. deviation = 0.0323 Å and maximum deviations of 0.0336 (13) and -0.0331 (13) Å for the N3 and N1 atoms, respectively. The Ni atom lies 0.0056 (13) Å out of the plane. The maximum deviations from the ideal geometry are manifested in the N1—Ni—N3 chelate angle of 83.28 (12)°. The pyridine molecule is inclined to the N_3_O donor set, forming a dihedral angle of 44.43 (6)°. The molecular structure resembles that of the *S*-methyl ester where the Ni atom is coordinated by Ph_3_P rather than pyridine (Guveli & Ulkuseven, 2011).

The most notable feature of the crystal packing is the formation of π—π interactions whereby the pyridine links alternating five- [inter-centroid distance = 3.4784 (16) Å, angle of inclination = 4.67 (14)° for symmetry operation: 1/2 + *x*, 1/2 - *y*, 2 - *z*] and six-membered [3.4633 (17) Å and 4.13 (13)° for -1/2 + *x*, 1/2 - *y*, 2 - *z*] chelate rings along the *a* axis, Fig. 2. Stacks assemble without specific interactions between them, Fig. 3.

### Experimental

Nickel acetate tetrahydrate (0.25 g, 1.0 mmol) was added to a solution of 1-(2-hydroxyphenyl)ethanone *S*-ethylisothiosemicarbazone hydrobromide (0.25 g, 1.0 mmol) in ethanol (10 ml). Three drops of pyridine was added to solution. The red solution was heated under reflux for 1 h. Orange prisms were deposited after 3 days, collected by filtration, washed with ethanol, and dried in air. *M*. pt: 421 K. Yield: 85%.

### Refinement

Carbon-bound H-atoms were placed in calculated positions [C—H = 0.95–0.99 Å, *U*_iso_(H) = 1.2–1.5*U*_eq_(C)] and were included in the refinement in the riding model approximation. Nitrogen-bound H-atom was refined with N—H = 0.88±0.01 Å and free *U*_iso_.

### Crystal data

**Table:**

[Ni(C_11_H_13_N_3_OS)(C_5_H_5_N)]	*F*(000) = 776
*M**_r_* = 373.11	*D*_x_ = 1.586 Mg m^−^^3^
Orthorhombic, *P*2_1_2_1_2_1_	Mo *K*α radiation, λ = 0.71073 Å
Hall symbol: P 2ac 2ab	Cell parameters from 2330 reflections
*a* = 7.2956 (4) Å	θ = 2.8–27.5°
*b* = 9.8463 (5) Å	µ = 1.39 mm^−^^1^
*c* = 21.7489 (11) Å	*T* = 100 K
*V* = 1562.33 (14) Å^3^	Prism, orange
*Z* = 4	0.35 × 0.10 × 0.05 mm

### Data collection

**Table:**

Agilent SuperNova Dual diffractometer with an Atlas detector	3584 independent reflections
Radiation source: SuperNova (Mo) X-ray Source	3130 reflections with *I* > 2σ(*I*)
Mirror monochromator	*R*_int_ = 0.037
Detector resolution: 10.4041 pixels mm^-1^	θ_max_ = 27.6°, θ_min_ = 2.8°
ω scan	*h* = −9→8
Absorption correction: multi-scan (*CrysAlis PRO*; Agilent, 2012)	*k* = −10→12
*T*_min_ = 0.790, *T*_max_ = 1.000	*l* = −28→24
6002 measured reflections	

### Refinement

**Table:**

Refinement on *F*^2^	Secondary atom site location: difference Fourier map
Least-squares matrix: full	Hydrogen site location: inferred from neighbouring sites
*R*[*F*^2^ > 2σ(*F*^2^)] = 0.040	H atoms treated by a mixture of independent and constrained refinement
*wR*(*F*^2^) = 0.075	*w* = 1/[σ^2^(*F*_o_^2^) + (0.0263*P*)^2^] where *P* = (*F*_o_^2^ + 2*F*_c_^2^)/3
*S* = 1.00	(Δ/σ)_max_ = 0.001
3584 reflections	Δρ_max_ = 0.45 e Å^−^^3^
213 parameters	Δρ_min_ = −0.39 e Å^−^^3^
1 restraint	Absolute structure: Flack (1983), 1501 Friedel pairs
Primary atom site location: structure-invariant direct methods	Flack parameter: −0.028 (16)

### Special details

**Table:**

Geometry. All e.s.d.'s (except the e.s.d. in the dihedral angle between two l.s. planes) are estimated using the full covariance matrix. The cell e.s.d.'s are taken into account individually in the estimation of e.s.d.'s in distances, angles and torsion angles; correlations between e.s.d.'s in cell parameters are only used when they are defined by crystal symmetry. An approximate (isotropic) treatment of cell e.s.d.'s is used for estimating e.s.d.'s involving l.s. planes.
Refinement. Refinement of *F*^2^ against ALL reflections. The weighted *R*-factor *wR* and goodness of fit *S* are based on *F*^2^, conventional *R*-factors *R* are based on *F*, with *F* set to zero for negative *F*^2^. The threshold expression of *F*^2^ > σ(*F*^2^) is used only for calculating *R*-factors(gt) *etc*. and is not relevant to the choice of reflections for refinement. *R*-factors based on *F*^2^ are statistically about twice as large as those based on *F*, and *R*- factors based on ALL data will be even larger.

### Fractional atomic coordinates and isotropic or equivalent isotropic displacement parameters (Å^2^)

**Table:**

	*x*	*y*	*z*	*U*_iso_*/*U*_eq_	
Ni	0.14271 (5)	0.31649 (4)	0.939044 (16)	0.00822 (10)	
S1	0.36781 (11)	0.04998 (8)	0.79776 (3)	0.01256 (17)	
O1	0.0483 (3)	0.4726 (2)	0.97176 (9)	0.0118 (5)	
N1	0.1406 (4)	0.3762 (3)	0.85785 (10)	0.0089 (5)	
N2	0.2056 (3)	0.2818 (3)	0.81453 (12)	0.0104 (6)	
N3	0.2446 (3)	0.1623 (3)	0.90504 (12)	0.0103 (6)	
H3n	0.302 (4)	0.099 (3)	0.9257 (13)	0.021 (10)*	
N4	0.1443 (4)	0.2372 (3)	1.01959 (10)	0.0089 (5)	
C1	−0.0040 (4)	0.5820 (3)	0.94155 (15)	0.0099 (6)	
C2	−0.0797 (4)	0.6887 (4)	0.97594 (14)	0.0129 (7)	
H2	−0.0866	0.6793	1.0193	0.015*	
C3	−0.1441 (4)	0.8058 (3)	0.94965 (13)	0.0131 (6)	
H3	−0.1943	0.8759	0.9745	0.016*	
C4	−0.1351 (4)	0.8210 (3)	0.88596 (13)	0.0149 (6)	
H4	−0.1789	0.9017	0.8671	0.018*	
C5	−0.0625 (4)	0.7188 (3)	0.85051 (14)	0.0138 (7)	
H5	−0.0596	0.7300	0.8071	0.017*	
C6	0.0081 (4)	0.5972 (3)	0.87607 (14)	0.0096 (7)	
C7	0.0840 (4)	0.4927 (3)	0.83555 (14)	0.0092 (7)	
C8	0.0990 (4)	0.5138 (3)	0.76748 (13)	0.0132 (7)	
H8A	0.2278	0.5071	0.7550	0.020*	
H8B	0.0517	0.6039	0.7568	0.020*	
H8C	0.0273	0.4442	0.7461	0.020*	
C9	0.2640 (4)	0.1738 (4)	0.84474 (14)	0.0102 (6)	
C10	0.3791 (5)	−0.0970 (3)	0.84806 (13)	0.0132 (7)	
H10A	0.4728	−0.0826	0.8803	0.016*	
H10B	0.2592	−0.1119	0.8682	0.016*	
C11	0.4295 (4)	−0.2193 (3)	0.80864 (15)	0.0190 (8)	
H11A	0.4229	−0.3020	0.8336	0.029*	
H11B	0.5544	−0.2081	0.7928	0.029*	
H11C	0.3437	−0.2264	0.7741	0.029*	
C12	0.0869 (4)	0.1084 (3)	1.02762 (14)	0.0111 (7)	
H12	0.0466	0.0583	0.9928	0.013*	
C13	0.0843 (4)	0.0461 (4)	1.08490 (15)	0.0188 (8)	
H13	0.0408	−0.0444	1.0892	0.023*	
C14	0.1455 (5)	0.1168 (4)	1.13532 (14)	0.0188 (8)	
H14	0.1469	0.0759	1.1749	0.023*	
C15	0.2050 (4)	0.2491 (4)	1.12707 (15)	0.0169 (8)	
H15	0.2485	0.3004	1.1611	0.020*	
C16	0.2005 (4)	0.3057 (4)	1.06906 (13)	0.0125 (7)	
H16	0.2391	0.3972	1.0641	0.015*	

### Atomic displacement parameters (Å^2^)

**Table:**

	*U*^11^	*U*^22^	*U*^33^	*U*^12^	*U*^13^	*U*^23^
Ni	0.01111 (18)	0.00732 (18)	0.00623 (17)	0.00083 (17)	0.00031 (17)	0.00024 (17)
S1	0.0188 (4)	0.0093 (4)	0.0096 (4)	0.0025 (4)	0.0027 (4)	−0.0010 (3)
O1	0.0203 (12)	0.0087 (12)	0.0064 (11)	0.0043 (10)	−0.0002 (9)	0.0011 (10)
N1	0.0105 (13)	0.0090 (13)	0.0072 (12)	−0.0001 (12)	0.0003 (12)	−0.0036 (10)
N2	0.0151 (13)	0.0081 (14)	0.0080 (13)	0.0013 (10)	0.0020 (10)	−0.0025 (11)
N3	0.0170 (14)	0.0078 (16)	0.0061 (13)	0.0031 (12)	−0.0001 (10)	−0.0010 (12)
N4	0.0096 (12)	0.0090 (13)	0.0081 (12)	0.0018 (12)	0.0051 (12)	0.0002 (10)
C1	0.0061 (14)	0.0127 (16)	0.0108 (15)	−0.0014 (11)	−0.0009 (14)	0.0025 (15)
C2	0.0140 (15)	0.0141 (16)	0.0106 (15)	−0.0003 (15)	−0.0003 (12)	−0.0032 (16)
C3	0.0139 (14)	0.0110 (15)	0.0144 (15)	0.0019 (15)	0.0006 (14)	−0.0033 (14)
C4	0.0167 (15)	0.0100 (15)	0.0179 (15)	0.0051 (17)	−0.0012 (14)	0.0035 (15)
C5	0.0164 (17)	0.0152 (18)	0.0097 (15)	0.0026 (14)	0.0003 (13)	0.0015 (14)
C6	0.0090 (16)	0.0104 (17)	0.0093 (15)	−0.0017 (13)	0.0011 (12)	−0.0011 (14)
C7	0.0066 (15)	0.0084 (16)	0.0126 (16)	−0.0021 (12)	−0.0020 (12)	−0.0019 (14)
C8	0.0187 (18)	0.0131 (17)	0.0077 (15)	0.0049 (14)	−0.0025 (13)	0.0011 (14)
C9	0.0082 (15)	0.0120 (17)	0.0104 (15)	−0.0008 (14)	−0.0010 (12)	−0.0025 (16)
C10	0.0174 (18)	0.0089 (15)	0.0132 (15)	0.0047 (14)	0.0009 (14)	0.0001 (13)
C11	0.0280 (19)	0.0109 (18)	0.0181 (18)	0.0032 (14)	0.0036 (15)	0.0003 (15)
C12	0.0129 (17)	0.0114 (17)	0.0090 (15)	−0.0011 (13)	0.0004 (13)	−0.0012 (14)
C13	0.0182 (18)	0.0155 (18)	0.0227 (19)	0.0041 (14)	0.0057 (14)	0.0061 (16)
C14	0.0195 (18)	0.025 (2)	0.0120 (16)	0.0090 (17)	0.0079 (16)	0.0119 (15)
C15	0.0157 (18)	0.025 (2)	0.0106 (17)	0.0043 (15)	−0.0012 (13)	−0.0015 (16)
C16	0.0138 (15)	0.0130 (16)	0.0107 (15)	0.0023 (13)	−0.0003 (12)	−0.0031 (16)

### Geometric parameters (Å, º)

**Table:**

Ni—O1	1.828 (2)	C5—C6	1.416 (4)
Ni—N1	1.861 (2)	C5—H5	0.9500
Ni—N3	1.845 (3)	C6—C7	1.464 (4)
Ni—N4	1.918 (2)	C7—C8	1.499 (4)
S1—C9	1.762 (3)	C8—H8A	0.9800
S1—C10	1.816 (3)	C8—H8B	0.9800
O1—C1	1.318 (4)	C8—H8C	0.9800
N1—C7	1.312 (4)	C10—C11	1.523 (4)
N1—N2	1.405 (3)	C10—H10A	0.9900
N2—C9	1.321 (4)	C10—H10B	0.9900
N3—C9	1.324 (4)	C11—H11A	0.9800
N3—H3n	0.871 (10)	C11—H11B	0.9800
N4—C16	1.334 (4)	C11—H11C	0.9800
N4—C12	1.347 (4)	C12—C13	1.389 (4)
C1—C2	1.403 (4)	C12—H12	0.9500
C1—C6	1.435 (4)	C13—C14	1.373 (5)
C2—C3	1.371 (4)	C13—H13	0.9500
C2—H2	0.9500	C14—C15	1.385 (5)
C3—C4	1.395 (4)	C14—H14	0.9500
C3—H3	0.9500	C15—C16	1.380 (4)
C4—C5	1.374 (4)	C15—H15	0.9500
C4—H4	0.9500	C16—H16	0.9500
			
O1—Ni—N3	178.07 (11)	C6—C7—C8	121.6 (3)
O1—Ni—N1	95.77 (11)	C7—C8—H8A	109.5
N3—Ni—N1	83.28 (12)	C7—C8—H8B	109.5
O1—Ni—N4	89.37 (10)	H8A—C8—H8B	109.5
N3—Ni—N4	91.65 (11)	C7—C8—H8C	109.5
N1—Ni—N4	174.40 (12)	H8A—C8—H8C	109.5
C9—S1—C10	102.80 (15)	H8B—C8—H8C	109.5
C1—O1—Ni	127.0 (2)	N2—C9—N3	121.8 (3)
C7—N1—N2	115.9 (2)	N2—C9—S1	114.1 (2)
C7—N1—Ni	129.0 (2)	N3—C9—S1	124.2 (3)
N2—N1—Ni	115.11 (19)	C11—C10—S1	107.6 (2)
C9—N2—N1	107.9 (2)	C11—C10—H10A	110.2
C9—N3—Ni	111.7 (2)	S1—C10—H10A	110.2
C9—N3—H3n	121 (2)	C11—C10—H10B	110.2
Ni—N3—H3n	125 (2)	S1—C10—H10B	110.2
C16—N4—C12	117.9 (3)	H10A—C10—H10B	108.5
C16—N4—Ni	122.2 (2)	C10—C11—H11A	109.5
C12—N4—Ni	120.0 (2)	C10—C11—H11B	109.5
O1—C1—C2	117.4 (3)	H11A—C11—H11B	109.5
O1—C1—C6	124.2 (3)	C10—C11—H11C	109.5
C2—C1—C6	118.4 (3)	H11A—C11—H11C	109.5
C3—C2—C1	122.9 (3)	H11B—C11—H11C	109.5
C3—C2—H2	118.6	N4—C12—C13	122.4 (3)
C1—C2—H2	118.6	N4—C12—H12	118.8
C2—C3—C4	119.2 (3)	C13—C12—H12	118.8
C2—C3—H3	120.4	C14—C13—C12	119.2 (3)
C4—C3—H3	120.4	C14—C13—H13	120.4
C5—C4—C3	119.8 (3)	C12—C13—H13	120.4
C5—C4—H4	120.1	C13—C14—C15	118.4 (3)
C3—C4—H4	120.1	C13—C14—H14	120.8
C4—C5—C6	122.6 (3)	C15—C14—H14	120.8
C4—C5—H5	118.7	C16—C15—C14	119.4 (3)
C6—C5—H5	118.7	C16—C15—H15	120.3
C5—C6—C1	117.1 (3)	C14—C15—H15	120.3
C5—C6—C7	119.7 (3)	N4—C16—C15	122.7 (3)
C1—C6—C7	123.2 (3)	N4—C16—H16	118.7
N1—C7—C6	120.8 (3)	C15—C16—H16	118.7
N1—C7—C8	117.6 (3)		
			
N1—Ni—O1—C1	2.0 (3)	O1—C1—C6—C7	−1.2 (5)
N4—Ni—O1—C1	−175.7 (2)	C2—C1—C6—C7	−179.9 (3)
O1—Ni—N1—C7	−1.2 (3)	N2—N1—C7—C6	178.1 (2)
N3—Ni—N1—C7	−179.5 (3)	Ni—N1—C7—C6	−0.5 (4)
O1—Ni—N1—N2	−179.8 (2)	N2—N1—C7—C8	−1.6 (4)
N3—Ni—N1—N2	1.9 (2)	Ni—N1—C7—C8	179.9 (2)
C7—N1—N2—C9	177.3 (3)	C5—C6—C7—N1	−176.1 (3)
Ni—N1—N2—C9	−3.9 (3)	C1—C6—C7—N1	2.0 (5)
N1—Ni—N3—C9	0.7 (2)	C5—C6—C7—C8	3.5 (4)
N4—Ni—N3—C9	178.3 (2)	C1—C6—C7—C8	−178.3 (3)
O1—Ni—N4—C16	−43.2 (2)	N1—N2—C9—N3	4.8 (4)
N3—Ni—N4—C16	135.2 (2)	N1—N2—C9—S1	−175.12 (19)
O1—Ni—N4—C12	136.6 (2)	Ni—N3—C9—N2	−3.5 (4)
N3—Ni—N4—C12	−45.0 (2)	Ni—N3—C9—S1	176.45 (17)
Ni—O1—C1—C2	177.51 (19)	C10—S1—C9—N2	−166.5 (2)
Ni—O1—C1—C6	−1.1 (4)	C10—S1—C9—N3	13.6 (3)
O1—C1—C2—C3	−178.0 (3)	C9—S1—C10—C11	168.3 (2)
C6—C1—C2—C3	0.7 (5)	C16—N4—C12—C13	0.1 (4)
C1—C2—C3—C4	0.1 (5)	Ni—N4—C12—C13	−179.7 (2)
C2—C3—C4—C5	0.1 (5)	N4—C12—C13—C14	−1.2 (5)
C3—C4—C5—C6	−1.2 (5)	C12—C13—C14—C15	1.0 (5)
C4—C5—C6—C1	1.9 (5)	C13—C14—C15—C16	0.2 (5)
C4—C5—C6—C7	−179.8 (3)	C12—N4—C16—C15	1.2 (5)
O1—C1—C6—C5	177.0 (3)	Ni—N4—C16—C15	−179.0 (2)
C2—C1—C6—C5	−1.6 (4)	C14—C15—C16—N4	−1.4 (5)

## References

[bb1] Agilent (2012). *CrysAlis PRO* Agilent Technologies, Yarnton, England.

[bb2] Ahmadi, M., Mague, T. J., Akbari, A. & Takjoo, R. (2012). Polyhedron, doi:10.1016/j.poly.2012.05.004.

[bb3] Brandenburg, K. (2006). *DIAMOND* Crystal Impact GbR, Bonn, Germany.

[bb4] Dilworth, J. R. & Hueting, R. (2012). *Inorg. Chim. Acta*, **389**, 3–15.

[bb5] Farrugia, L. J. (1997). *J. Appl. Cryst.* **30**, 565.

[bb6] Flack, H. D. (1983). *Acta Cryst.* A**39**, 876–881.

[bb7] Guveli, S. & Ulkuseven, B. (2011). *Polyhedron*, **30**, 1385–1388.

[bb8] Sheldrick, G. M. (2008). *Acta Cryst.* A**64**, 112–122. 10.1107/S0108767307043930 18156677

[bb9] Westrip, S. P. (2010). *J. Appl. Cryst.* **43**, 920–925.

